# The Extraction of Clear Lenses for Myopia

**Published:** 1894-06

**Authors:** H. E. Stafford

**Affiliations:** New York


### THE EXTRACTION OF CLEAR LENSES FOR MYOPIA. REPORT OF FIVE CASES.*

By H. E. STAFFORD, M.D., New York.

*Mr. President and Gentlemen of Georgia State Medical Association:*

Through the courtesy of several of your members I am invited to read a paper before you.

Not having had sufficient time to prepare an extensive article, I thought the report of five operations for myopia might prove interesting.

Previous to describing the operations, I will relate the circumstances which lead me to believe that the extraction of the lens, whether clear or not, would arrest the progress of myopia.

Some years ago I had a case of incipient cataract of both eyes, in a woman about thirty-two years old. The opacity at that time was not extensive enough to prevent a fair examination of the fundus.

The ophthalmoscope showed large posterior ectasia, fluid vitreous, floating opacities, about twenty dioptries of myopia in the right eye, and eighteen dioptries in the left.

Glasses of the accepted strength which, if I remember rightly, were twenty and eighteen D., were prescribed and improved the vision very much.

Patient returned in about ten months, with almost complete opacity of the lenses. I was entreated by the patient to remove the lens from the worst eye. I assured her that in all probability the hyaloid membrane would be ruptured, and the eye destroyed by total loss of vitreous; still she insisted that as she was almost sightless and would be totally blind in but a short time, she would much prefer taking the risk of failure in the operation, since the results in case of failure could be no worse.

Yielding to her determination to hazard a chance, I operated, and greatly to my surprise, did not lose vitreous.

*Read before Medical Association of Georgia, April, 1894.

After the right eye had healed I then operated on the left. The result in this eye was as good as the other. If I remember correctly, the vision was almost normal with a–11 in the right eye and –9 in the left.

This case was under observation about a year and a half before I realized the fact that the myopia had not increased. This caused me to believe that the removal of the lens and wearing of the correcting glasses had arrested the disease.

Case 1.—Progressive myopia, twenty-five D. one eye, sixteen D. the other, duration three years.

Large posterior staphyloma, fluid vitreous and floating opacities in both eyes. Lenses perfectly clear. Patient complained bitterly of our inability to arrest the progress of the disease, also of the wearing of such heavy glasses. I told him the results obtained in the cataract extraction, and expressed my belief that the removal of the clear lens would assist in arresting the elongation of the eyeball. At the same time I fully explained to him the great danger attending the operation. He also insisted, and being emboldened by the success in the other case, I assented.

Having a clear lens to extract, and being fully imbued with the popular superstition regarding the extraction of unripe lenses, I hesitated as to the method of operation.

I was tempted to artificially ripen the lens, but decided to do an ordinary extraction. This was done without the speculum (so as to avoid the loss of valuable time should occasion demand the rapid closure of the lids) without an iridectomy, lacerating the capsule of the lens with the point of the knife as it passed across the anterior chamber. The lenses were delivered by the lids for the same reason that the speculum was discarded. The whole operation was completed without loss of vitreous.

I found the clear lens as easy to remove as the opaque ones, and a careful examination proved that it had retained its continuity. After instillation of a drop of a one grain to the ounce solution of eserine, the eye was treated as usual after extraction and healed rapidiy.

Vision R. E. before operation 20-200–w–20 D., after operation 20-40–w–15 D.

Saw patient ten months afterwards, no increase of myopia in this eye. Left eye decided increase of the myopia. Patient insisted upon operation, which was performed in the same manner as the other.

Before operation, ophthalmoscope showed 30 D. myopia, best vision 15-200–w–20 D. After operation 20-50–w–20 D.

CASE 2.—Woman, twenty-five years old. Progressive myopia, duration four years.

R. E. ophthalmoscope showed 20 D. myopia. Posterior ectasia floating bodies, fluid vitreous, vision 20-70–w–20 D. Vision after operation 20-40- w–11 D.

L. E. ophthalmoscope eighteen D. myopia, medium sized ectasia, fluid vitreous. Vision 20-50–w–18 D. After operation 20-30–w–7 D.

In this eye some of the lens substance remained. It being a very small amount, and fearful of loss of vitreous should I manipulate the eye in attempting its removal, I allowed it to remain. Atropine was instilled daily, and I saw the small portion become opaque, swell to about the size of a small duck shot, and be absorbed without reaction.

This case I saw shortly before leaving New York and found no increase of the myopia.

CASE 3.—This was not my own, but was brought to me after operation. Woman, about forty years old. The lens of left eye had been needled before extraction to ripen it. It had swelled greatly and produced considerable reaction, necessitating immediate removal. This was done without iridectomy.

The results in this case were not at all satisfactory. The capsules were thickened, opaque, and a tough dense membrane bound the iris to them.

I advised a secondary needling, which was done, but capsules again closed. The only recourse left, apparently, is the removal of capsules and membrane by hook or forceps, an operation I would not care to perform with fluid vitreous.

The results in these cases fully convince me that the operation should be done without attempting artificial ripening. Indeed, in any case of partial opacity of the lens, I do not hesitate to do the usual extraction, finding absolutely no difference in the results.

I know that this operation is dangerous, yet it has demonstrated a decided influence in arresting the progress of this disease.

Not knowing that this operation has never been performed before with this object in view, I present this report to you, trusting that the results may prove as practical in the hands of others as in my own.

